# Effect of Photodynamic Therapy on Halitosis: A Systematic Review of Randomized Controlled Trials

**DOI:** 10.3390/s22020469

**Published:** 2022-01-08

**Authors:** Pamella de Barros Motta, Lara Jansiski Motta, Thalita Molinos Campos, Marcela Leticia Leal Gonçalves, Elaine Marcílio Santos, Ana Luiza Cabrera Martimbianco, David José Casimiro de Andrade, Raquel Agnelli Mesquita-Ferrari, Kristianne Porta Santos Fernandes, Anna Carolina Ratto Tempestini Horliana, Sandra Kalil Bussadori

**Affiliations:** 1 Postgraduation Program in Biophotonics Applied to Health Sciences, Universidade Nove de Julho (UNINOVE), Sao Paulo 01504-001, SP, Brazil; pamellabmotta@gmail.com (P.d.B.M.); larajmotta@terra.com.br (L.J.M.); thalita.molinos@gmail.com (T.M.C.); marcelalleal@hotmail.com (M.L.L.G.); raquel.mesquita@gmail.com (R.A.M.-F.); kristianneporta@gmail.com (K.P.S.F.); annacrth@gmail.com (A.C.R.T.H.); 2 Postgraduation Program in Health and Environment, Universidade Metropolitana de Santos (UNIMES), Santos 11045-002, SP, Brazil; elaine.marcilio@unimes.br (E.M.S.); analuizacabrera@hotmail.com (A.L.C.M.); 3 School of Dentistry, University of Porto, 4099-002 Porto, Portugal; casimiroandrade@gmail.com

**Keywords:** photodynamic therapy, halitosis, laser, systematic review

## Abstract

Background: This systematic review aimed to assess the effectiveness and safety of aPDT for the treatment of halitosis. Methods: Search strategies were conducted in October 2021 without language or data restrictions, on the following databases: MEDLINE, EMBASE, CENTRAL, LILACS and BBO, as well as a manual search. Randomized clinical trials (RCTs) with parallel design were considered for inclusion, assessing individuals (adolescents and adults) with a clinical diagnosis of halitosis treated with photodynamic therapy (aPDT). Primary outcomes assessed were halitosis measurements, adverse events and quality of life. The risk of bias for each included study was evaluated with the Cochrane Risk of Bias tool and the certainty of the body of the evidence was assessed with the GRADe approach. Results: Six RCTs (total of 225 participants) were included and due to clinical diversities it was not possible to group the outcome data in meta-analyses. Based on very low-certainty evidence (GRADE) the results showed that, when compared to tongue scraper, aPDT seems to promote a little to no difference in reducing halitosis and in the microbiological analysis. No adverse events were reported. Considering aPDT combined with tongue scraper, better outcome results were observed when compared to tongue scraper alone. Conclusions: Based on very low-certainty evidence, the findings of this review are uncertain about the effects of aPDT for halitosis control. Further RCTs with higher number of participants and long term assessments need to be conducted to support the use of this intervention. The protocol was registered in the PROSPERO database (number: CRD42020215319) on 19 November 2020—retrospectively registered.

## 1. Introduction

Halitosis is a term that consists of any unpleasant odor emanating from the oral cavity, the source of which may be local or systemic [1]. This alteration in mouth odor is the third major cause of the search for oral treatment [2]. Anaerobic bacteria are identified as the main cause of halitosis. These microorganisms produce sulfur-rich gases, which cause the presence of smell. Three volatile sulfur compounds (VSCs) are related to halitosis: hydrogen sulfide (H_2_S), methylmercaptan (CH_3_SH), and dimethyl sulfide (CH_3_SCH_3_) [3,4,5,6]. The high concentration of mucin in the saliva aids the adhesion of anaerobic microorganisms and epithelial cells in the posterior third of the lingual dorsum [7]. This biofilm is called tongue coating [8], and it is the most common cause of halitosis. Hydrogen sulfide (H_2_S) is the main gas liberated by bacteria from the tongue surface [9].

The different diagnostic methods for halitosis include a clinical assessment, known as the organoleptic test, which is a subjective method that consists of smelling the air exhaled from the mouth and quantifying the odor with the use of a scale. Halitosis can also be measured with a sulfide monitor, as described by Guedes et al. [10]; this method is an alternative to the organoleptic test and has high sensitivity and specificity [10]. Gas chromatography is the most appropriate method for the diagnosis of halitosis of any origin, as this method measures the three main sulfur gases [11,12,13]. The prevalence of this condition is high, with percentages above 50% found in articles [14]. 

Many interventions to improve halitosis have already been tested, but there is no evidence of superiority between them [15]. Existing treatments have disadvantages, such as staining of mucous membranes and teeth [16,17]. Conventional treatments for the control of halitosis basically consist of the use of toothpastes and mouthwashes with bactericidal substances, the correct use of tongue scrapers, the removal of dental caries lesions, the treatment of periodontal diseases, and the control of possible cases of xerostomia [12,13]. The tongue scraper has the disadvantage of causing excessive excoriation of the surface of the tongue, which is also a discomfort when eating acidic or bitter foods after excoriation of the tongue [18]. Oral hygiene behavior (OHB) is a very efficient complementary method [19]. 

Antimicrobial photodynamic therapy (aPDT) [18,20,21] has been tested in an attempt to treat halitosis [14,22]. It involves a photosensitizing agent, which produces free oxygen radicals (type I reaction) and singlet oxygen (type II reaction) in the presence of light, thereby destroying the cell wall of bacteria and causing cell death. This approach avoids both the occurrence of resistant bacteria and harm to the adjacent tissues, as the antimicrobial effect is confined to the areas covered by the photosensitizer and irradiated with light, acting quickly on the target microorganisms [18,20]. For halitosis, the main etiological factor of which is anaerobic bacteria, this therapy has achieved positive results regarding the reduction in hydrogen sulfide as well as a reduction in the bacterial load on the dorsum of the tongue, using a laser at a red wavelength and methylene blue. The advantages of this alternative approach are the reduction in damage to the tissues, the avoidance of bacterial resistance, and the development of a treatment protocol for halitosis that may be effective and lasting by eliminating the anaerobic bacteria related to this condition [18,20,23]. However, it is important to consider that even with immediate positive results, it was demonstrated that after 7 days, the participants returned to the initial halitosis values, which reinforces that the treatment for halitosis could be accompanied by oral hygiene behavior [18]. This probably occurs because bacteria residing in other niches of the oral cavity could recolonize the back of the newly treated tongue [24].

With the emergence of alternative treatments, such as aPDT, it is essential to analyze the level of evidence and the results of these novel protocols to assist dentists in the management of halitosis. Thus, the aim of the present study is to perform a systematic review of randomized controlled clinical trials that used aPDT protocols for the treatment of halitosis.

A large number of studies are led in the health field and need to be summarized [25] in systematic reviews. Such studies assist in the development of novel protocols for daily clinical practice [26], as decisions based on a single study could lead to errors. Moreover, this type of study enables an appraisal of the level of evidence found in articles and the effectiveness of the proposed treatments [27].

Halitosis is considered an important social drawback that affects interpersonal relations. In addition to raising concerns regarding the physical health of the individual, this condition can generate psychological problems and constitute a social barrier [28]. A systematic review enables the reproducibility of treatments proposed in clinical trials and therefore aggregates scientific evidence to these protocols. This type of study also assists in the development of new questions for future studies [29]. In the case of aPDT for the treatment of halitosis, it is necessary to investigate whether this therapy is more effective when used alone or whether it should be combined with another treatment modality. Therefore, the present systematic review aimed to assess the effectiveness and safety of aPDT for the treatment of halitosis.

## 2. Materials and Methods

This systematic review follows the methodological recommendations of the Cochrane Handbook for Systematic Reviews of Interventions [30] and the report guidance of PRISMA [31]. The protocol registration was made in International prospective register of systematic reviews (PROSPERO) (retrospective register, under the number CRD42020215319).

### 2.1. Eligibility Criteria

#### 2.1.1. Types of Studies

We considered for inclusion only randomized clinical trials (RCTs) with parallel design.

#### 2.1.2. Types of Participants

Individuals (adolescents and adults) with a clinical diagnosis of halitosis.

#### 2.1.3. Types of Interventions and Comparators

We included RCT that assessed the use of photodynamic therapy at any therapeutic parameter, dose and duration, compared with placebo, no intervention or another active treatment such as tongue scraper. If a co-intervention was administered in combination with aPDT, the study was included only if this co-intervention was given to both groups.

### 2.2. Outcomes Assessed

#### 2.2.1. Primary

Halitosis measurements: hydrogen sulfide (H_2_S) gas, measured in parts per billion (ppb), for example by a gas chromatography test.

Adverse events (such as discomfort, gagging sensation, among others).

Quality of life (measured for example OHIP 14).

#### 2.2.2. Secondary

Microbiological analysis, measured in colony forming unit (CFU/mL).

Patient-reported halitosis perception (measured as reported by the included studies).

#### 2.2.3. Search Strategies

Broad and sensitive search strategies were performed on each of the following electronic databases: Medical Literature Analysis and Retrieval System Online (MEDLINE, via Pubmed), Cochrane Central Register of Controlled Trials (CENTRAL, via Wiley), Excerpta Medica database (EMBASE, via Elsevier), BBO (Bibliografia Brasileira de Odontologia) (both via BVS—Biblioteca Virtual em Saúde) and LILACS (Literatura Latino-Americana e do Caribe em Ciências da Saúde). An additional manual search was conducted in the reference lists of the relevant studies. The searches were run on 12 January 2021 and updated on 28 October 2021, without date and language restrictions. The detailed search strategies for each database were presented in the Supplementary Materials Table S1. 

### 2.3. Study Selection Process and Data Extraction

The references screened from the search strategies were selected by two independent authors through the Rayyan platform [32]. After the removal of duplicates, the references were analyzed based on the eligibility criteria by titles and abstracts. Those studies that fulfil the eligibility criteria were assessed in a second stage by full texts in order to confirm inclusion or exclusion. A third author solved the disagreements.

The data extraction procedure was also performed by two independent authors, using a standard form through Microsoft Excel^®^. Data that were extracted were: year of publication, number of patients, wavelength, photosensitizer, pre-irradiation time, energy, power, number of points irradiated, application time per point, number of sessions, and follow-up time. When necessary, the RCT authors were contacted for additional information.

### 2.4. Methodological Quality of the Included Studies

The risk of bias of each included study was assessed using the Cochrane Risk of Bias tool [30], which is composed of seven domains: random sequence generation, allocation concealment, blinding of participants and personnel, blinding of outcome assessors, incomplete outcome data, selective reporting, and other sources of bias. As recommended, we performed an outcome-level assessment for the domains: blinding of participants and researchers, blinding of outcome evaluators, incomplete outcome data. Each domain was judged according to the risk of bias as high, low, or unclear.

### 2.5. Data Synthesis

We planned to conduct meta-analyses using the software Review Manager 5.4.1, when the data from the included studies were available and homogenous. However, due to the lack of numerical data and the clinical diversity between included studies, the outcome data were described narratively and the estimated effects were calculated when possible (data availability). For continuous data, mean differences (MDs) were calculated between treatment groups. For dichotomous data, we planned to use the number of events in the intervention and control groups of each study to calculate risk ratios (RRs). A 95% confidence interval (CI) was considered. We planned to identify the methodological and clinical diversity of the studies if meta-analyses were conducted, as well. The presence of statistical heterogeneity among studies would be assessed by Chi² test, and its extension by the I2 test (I2 > 50% indicates substantial heterogeneity).

### 2.6. Assessing the Certainty of the Evidence 

The GRADE approach (Grading of Recommendations, Assessment, Development and Evaluations) was used to evaluate the certainty of the overall body of evidence [33]. The GRADE encompasses five domains to downgrade the certainty of the evidence from RCTs (methodological limitations, inconsistency, imprecision, indirectness and publication bias). A summary of the findings table using the GRADEpro GDT software was generated, and the reasons to downgrade the certainty of the evidence were detailed.

## 3. Results

**Figure 1:**
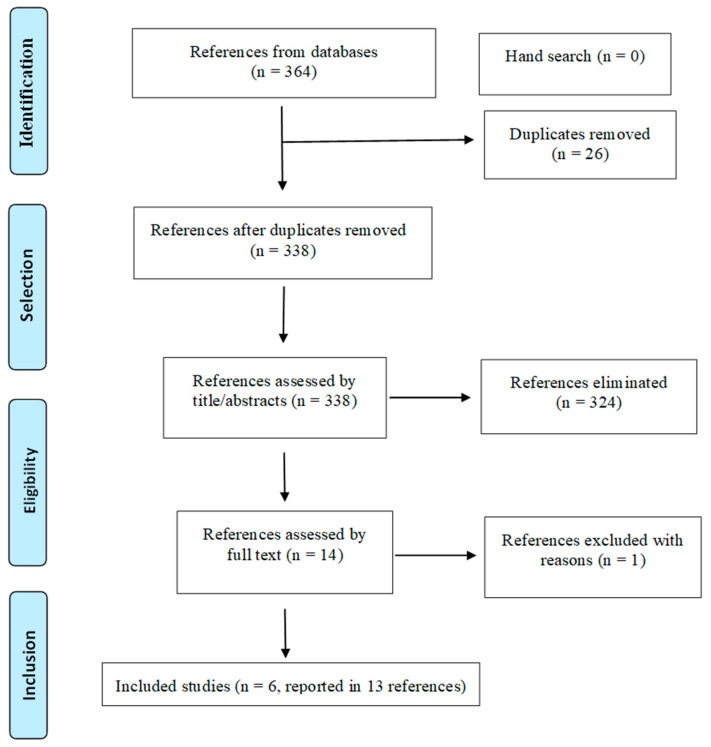
PRISMA flowchart of the study selection process.

Database search identified 364 references. After removing 26 duplicates, 338 were screened by title and abstract and 324 were excluded for not fulfilling the inclusion criteria. Fourteen studies were analysed in full text and one was excluded due to wrong study design [34]. Thus, six randomized clinical trials (RCTs) (reported in 13 references) [18,20,24,34,35,36,37,38,39,40,41,42,43,44] were included in this systematic review (Figure 1).

### 3.1. Characteristics of the Included Studies 

The six RCTs involved a total of 255 participants and assessed halitosis reduction after the application of aPDT. They were published between 2016 and 2021 and were led in two countries: Brazil and Saudi Arabia. Table 1 detailed their main characteristics.

### 3.2. Methodological Quality Assessment

**Figure 2:**
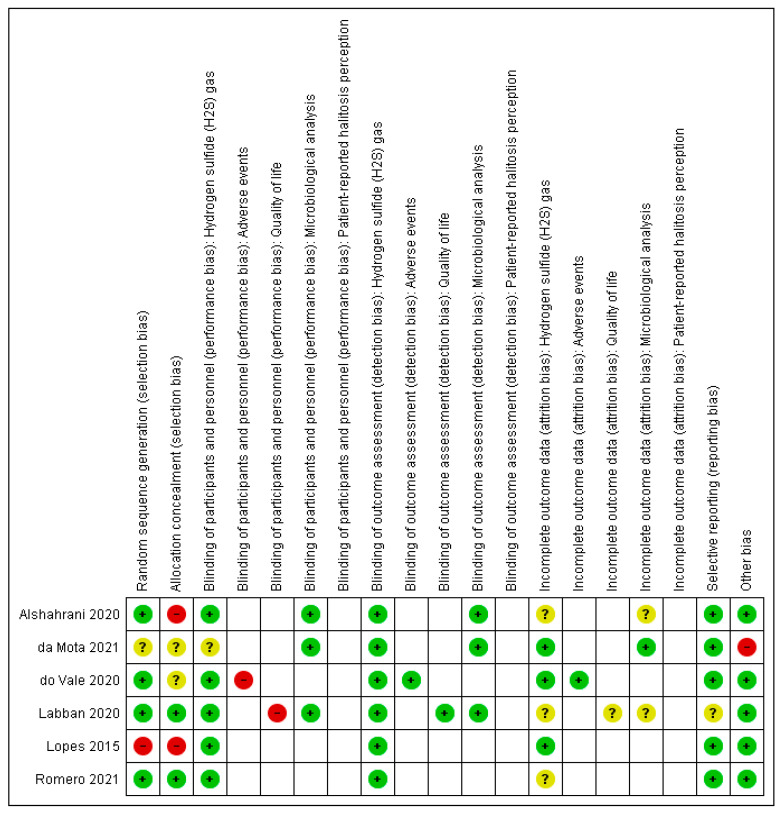
Risk of bias summary: review authors’ judgments about each risk of bias item for each included study.

Figure 2 presented a summary of the risk of bias for each domain for each included study. Only one study [36] presented an inadequate random sequence generation and allocation concealment and was rated as having a high risk of selection bias. Another study [41] presented a high risk of bias for allocation concealment because of the use of a non sealed envelopes to maintain the concealment of the random sequence. One study [44] did not provide information on both selection domains and was considered as having an unclear risk of selection bias. Regarding performance bias, two studies [42,43] assessed outcomes that could be compromised by the lack of participant blinding, occurring given the nature of the interventions including different procedures. These studies were judged as high risk of bias for the subjective outcomes. All included studies presented a low risk of detection bias because the outcomes assessed could not be influenced by the lack of outcome assessor blinding. Three studies [24,41,42] did not provide any information on losses of participants and were, therefore, classified as unclear risk of attrition bias. One study [42] had the clinical trial register not found and was considered with an unclear risk of reporting bias. Ultimately, one study [44] did not present the baseline characteristics of the participants and was classified as high risk of bias for the other sources of bias domain.

### 3.3. Effects of Intervention

#### 3.3.1. Comparison 1. Antimicrobial Photodynamic Therapy (aPDT) versus Tongue Scraper

##### Hydrogen Sulfide (H_2_S) Gas Measurement

Five RCTs assessed the reduction in halitosis, which was determined by the measurement of H_2_S using the OralChromaTM device [24,36,41,43,44]. Considering the clinical differences and the lack of some numerical data it was not possible to group the results in meta-analysis. Thus, the estimated effects were reported individually on Table 2.

##### Adverse Events

Only one RCT [43] (40 participants) assessed the presence of adverse events during the study and showed that most of the participants in the tongue scraper group reported discomfort or a gagging sensation during the procedure. No adverse events were observed in the aPDT group (very low-certainty evidence).

##### Microbiological Analysis

Three RCTs conducted microbiological analysis but it was not possible to conduct a meta-analysis due to the lack of numerical data; thus, the findings of each study were described individually. One RCT [44] reported no statistical difference between aPDT and tongue scrape for the following bacteria investigated: Porphyromonas gingivalis and Tannerella forsythia (*p* > 0.05, 30 participants). However, the analysis of Treponema denticola identified a statistical difference in favour of aPDT after 7 days (*p* = 0.004) and 14 days (*p* = 0.006) post-treatment. Another RCT [36] reported no statistical difference between groups (*p* = 0.05, 30 participants) regarding microbiological examination. Lastly, one RCT reported no difference between groups [41].

#### 3.3.2. Comparison 2. Antimicrobial Photodynamic Therapy (aPDT) plus Tongue Scraper versus Tongue Scraper

##### Hydrogen Sulfide (H_2_S) Gas Measurement

Three studies evaluated this outcome but the lack of numerical data precluded performing a meta-analysis and data were described individually:

Labban et al. (2020) [42]: the authors reported significant improvement in H_2_S concentration in the aPDT plus tongue scraper group when compared with the tongue scraper group (median 148 versus 0 ppb; *p* = 0.001, 40 participants).

Alshahrani et al. (2020) [41]: the authors reported a reduced H_2_S concentration in favour of aPDT plus tongue scraper (median [IQT] 0 [0] versus 65 ppb, *p* = 0.0001, 29 participants).

Lopes et al. (2015) [36]: the authors reported a reduced H_2_S concentration in favour of aPDT plus tongue scraper (median [IQT] 0 [0] versus 53 [7] ppb, *p* = 0.0003, 29 participants).

##### Quality of Life

Estimated effect from one RCT showed an imprecise result on the improvement in the quality of life measured by the OHIP-14 summary scores. There was a wide confidence interval compatible with both a decrease and an increase in the score, and a small sample size (MD 20.05, 95% CI−53.22 to 93.23, 40 participants) [43].

##### Microbiological Analysis

One RCT reported a statistically significant reduction in Porphyromonas gingivalis with aPDT only after 5 days of treatment (*p* < 0.05, 40 participants) (no numerical data was provided) [42]. Another RCT reported a statistical bacterial reduction in the aPDT plus tongue scrape group when compared to tongue scrape (*p* = 0.0003, 29 participants) [36]. Lastly, one RCT reported no difference between groups [41].

##### Certainty of the Evidence

The certainty of the body of evidence was evaluated with the GRADE approach for primary outcomes assessed in the main comparison: antimicrobial photodynamic therapy (aPDT) versus tongue scraper. The evidence was rated as very low due to methodological limitations and imprecision (wide confidence interval, small sample size and few events). This indicates very little confidence in the effect estimate: the true effect is likely to be substantially different from the estimate of effect. The summary of findings in Table 3 presented the assessment and judgements.

## 4. Discussion

Halitosis is the third major cause of the search for oral treatment. Thus, it is relevant to address this problem, which can exert a considerable impact on quality of life [2]. The main cause of halitosis is gas (volatile sulfur compounds) produced by bacteria found in coated tongue. Therefore, treatment consists of the control of these bacteria through mechanical removal, chemical removal, or cell death due to phototoxicity [44]. Tongue scraping could be easily carried out by patients themselves and is widely recommended, but it is little practiced due to discomfort, as it can cause nausea, or lack of awareness regarding its use. In addition, studies have shown that self-cleaning of the tongue alone is not completely efficient for reducing halitosis and it should be associated with in-office treatments, such as periodontal ones. Consequently, alternative forms of treatment, such as aPDT, which can be performed in an office, and is less aggressive to the papillae (that can be hurt during the scraping process), are being researched. 

The objective of the present study was to evaluate the efficacy of aPDT at reducing halitosis in comparison to other therapies. The authors of this review believe that alternative therapies, such as the one studied herein, have an advantage over tongue scrapers, as the mechanical removal of coated tongue can cause damage to the lingual papillae. In all articles analyzed, aPDT seems to be effective at achieving an immediate reduction in halitosis. Do Vale et al. [43] and Romero et al. [24] found that aPDT was more effective for the treatment of halitosis when compared to the use of a tongue scraper, whereas Mota et al. [44], Lopes et al. [36] and Alshahrani [41] found that this therapy was more effective when combined with the mechanical removal of coated tongue.

The majority of studies used a red laser (λ = 660 nm) as the energy source and the photosensitizer was methylene blue at a concentration of 0.005%. The predominant pre-irradiation time was five minutes, but Romero et al. [24] used a pre-irradiation time of one minute and found that the treatment was effective with this shorter time. The most used energy was 9 J with a power of 100 mW at six points distributed on the dorsum of the tongue. Treatment was performed in a single session in all articles. The researchers who performed follow-up after seven days reported the return to initial H_2_S concentrations [24,43] whose halitosis remained low until the seventh day. These two authors evaluated edentulous patients, and prosthesis were also cleaned. Interestingly, patients were undergoing fixed orthodontic treatment. Alshahrani 2020 [41] also maintains low concentrations of H_2_S.

The primary outcome in the majority of articles included in the present review was a reduction in halitosis, which was determined based on the measurement of H_2_S using the OralChroma device. The studies that employed this device reported a reduction in the concentration of H_2_S immediately after the application of aPDT. This device employs gas chromatography for the determination of concentrations of volatile sulfur compounds produced by anaerobic bacteria, which is the main cause of halitosis [36]. The authors used a H_2_S concentration ≥112 ppb for the determination of halitosis. OralChroma is currently considered the gold standard. The diagnosis was previously performed using the organoleptic method, which has fallen into disuse because of the subjectivity of the evaluation.

All researchers who used the OralChroma device followed the manufacturer’s instructions and obeyed the following sequence: the participants were instructed to avoid spicy foods, alcohol, coffee, chewing gum, and mouthwash. On the day of the reading, the participants needed to fast for at least two hours and rinsed their mouths with cysteine (10 mM).

It was not possible to perform a meta-analysis (or sensitivity analysis) for the outcome of the research question of this systematic review. Despite being well designed, the studies showed clinical heterogeneity, especially in relation to the characteristics of the population such as different age range. Literature shows the occurrence of halitosis at all ages, but the causes and habits can change the characteristics in different age groups. In view of the heterogeneity of the studies, it was decided to carry out only the qualitative analysis of the studies. The results of the present systematic review show uncertain evidence about the effects of antimicrobial photodynamic therapy compared to the tongue scraper. The results seem to present better effects with the combination of both methods (aPDT and tongue scraper) when compared to the tongue scraper. However, since the available evidence was classified as very low certainty by the GRADE approach, further studies should be conducted to support these findings and to establish the number and periodicity of sessions needed to achieve the complete resolution of this problem.

The predominant use of methylene blue is related to the wavelengths employed by these researchers, which ranged from 655 to 660 nm (red laser). Mota et al. [44], Lopes et al. [36], and Vale et al. [43] applied the photosensitizer and waited for five minutes of pre-irradiation time, and Romero et al. [24] waited only one minute.

The parameters used for aPDT were quite similar among the studies, as the majority were performed by the same research group. All studies used red laser. Mota et al. [44], Lopes et al. [36], and Vale et al. [43] and Romero et al. [24] and used an energy of 9 J and power of 100 mW at six points on the dorsum of the tongue for 90 s per point. All papers in the present review performed a single session of aPDT.

Mota et al. [44], Lopes et al. [36] and Alshahrani 2020 [41] conducted similar studies, in which three groups were compared: (1) treatment with aPDT; (2) treatment with a tongue scraper; and (3) combination of aPDT and tongue scraper. In the study by Lopes et al. [36], the reduction in the concentration of H_2_S was 97% in Group 1, 88.6% in Group 2, and 100% in Group 3. Additionally, in the Alshahrani 2020 [41] study, Group 1 (aPDT) decreased 95%, Group 2 (TS) 89,4% and Group 3 (aPDT + TS) 100% in 15 days. Mota et al. [44] did not provide numerical data.

Do Vale et al. [43] and Romero et al. [24] allocated the participants into two groups: experimental (treatment with aPDT) and control (treatment with tongue scraper). In the study by do Vale et al. [43], the authors found a reduction in halitosis after both treatments. However, significant differences between groups were found immediately after treatment and at the seven-day follow-up, with greater reductions in hydrogen sulfide concentrations in the group treated with aPDT.

Lopes et al. [36] found that aPDT was effective at achieving an immediate reduction in halitosis and therefore constitutes a treatment option for this condition that does not harm the papillae, as occurs in conventional treatment with a tongue scraper. However, the authors found that the application of 90 s per point at six points on the dorsum of the tongue caused certain discomfort among the patients and suggested further studies to test different energies. Do Vale et al. [43] conducted a study with patients who wore complete dentures. The authors concluded that aPDT seems to be effective at reducing H_2_S immediately after treatment and that this effect was maintained at the seventh day follow-up. Laban et al. [42] concluded that antimicrobial PDT seems to help in reducing H_2_S concentration and improving quality of life in elderly patients wearing dentures. There also a reduction in P. gingivalis that occurred only in the short-term follow-up. Da Mota et al. [44] concluded that aPDT using a red LED and 0.005% methylene blue caused an immediate reduction in halitosis, but the effect was not maintained after 7, 14, or 30 days. Additionally, they found no reduction in the number of bacteria investigated or the quantification of universal 16S rRNA. Romero et al. [24] reinforces the oral hygiene behavior associated with aPDT or tongue scraper was not able to reduce halitosis after 90-day follow-up. Despite halitosis remaining higher than 112 ppb in all follow-up periods, the mean values remain two- or three-fold smaller than baseline values. Future studies should include other oral hygiene behavior to achieve better results in the treatment of halitosis. Alshahrani [41] concludes that PDT along with tongue scraping showed immediate reduction in H_2_S and reduction in oral pathogens in adolescent patients undergoing fixed orthodontic treatment for 15 days.

## 5. Conclusions

The results of the present systematic review show that antimicrobial photodynamic therapy administered alone seems to be an effective treatment for the control of halitosis, achieving better results than the sole use of a tongue scraper. Considering the small number of participants in the included studies and some methodological limitations identified, future randomized clinical trials are still necessary, with higher sample size and long-term outcome assessments to provide a confident guidance for decision-making.

## Figures and Tables

**Table 1:** Main characteristics of included studies.

Author/Year and Country	Population	Intervention	Comparators	Outcomes of Interest	Follow-Up	Funding Sources
Da Mota 2021 [44] Brazil	*n* = 45 Age18 and 25 years Sex 73% women H_2_S > 112 ppb	Photodynamic therapy (660 nm + 0.005% methylene blue)	Tongue scraper	- Hydrogen Sulfide (H_2_S) (ppb) - Microbiological analysis (PCRq)	Immediately after, 7, 14 and 30 days	The São Paulo Research Foundation National Council for Scientific and Technological Development
Lopes 2016 [36] Brazil	*n* = 45 Age 13 to 18 years Sex 26% women H_2_S > 112 ppb.	Photodynamic therapy (660 nm + 0.005% methylene blue)	Tongue scraper ____________ Photodynamic therapy (660 nm + 0.005% methylene blue) + Tongue scraper	- Hydrogen Sulfide (H_2_S) (ppb) - Microbiological analysis (CFU/mL)	Immediately after	State of São Paulo Research Assistance Foundation
Do Vale 2021 [43] Brazil	*n* = 40 Mean age 66.95 years Complete upper and lower dentures Sex 77% women H_2_S > 112 ppb.	Photodynamic therapy (660 nm + 0.005% methylene blue)	Tongue scraper	- Hydrogen Sulfide (H_2_S) (ppb) - Adverse events	Immediately after, 7 days	No funding sources
Romero 2021 [24] Brazil	*n* = 40 Mean age 34 years Sex 76% women H_2_S > 112 ppb.	Photodynamic therapy (660 nm + 0.005% methylene blue)	Tongue scraper	- Hydrogen Sulfide (H_2_S) (ppb)	Immediately after, 7, 90 days	No funding sources
Laban 2020 [42] Saudi Arabia	*n* = 40 Mean age 67.16 years Sex 55% women Complete upper and lower dentures H_2_S > 112 ppb.	Photodynamic therapy (660 nm + 0.005% methylene blue) + tongue scraper + full mouth disinfection + and adjunctive	Tongue scraper + full mouth disinfection	- Hydrogen Sulfide (H_2_S) (ppb) - Quality of life (measured by OHIP 14) - Microbiological analysis (CFU/mL)	After 5, 15 and 30 days	No funding sources
Alshahrani 2020 [41] Saudi Arabia	*n* = 45 12–17 years Sex 35% women undergoing fixed orthodontic treatment H_2_S > 112 ppb	Photodynamic therapy (660 nm + 0.005% methylene blue)	Tongue scraper ___________ Photodynamic therapy (660 nm + 0.005% methylene blue) + Tongue scraper	- Hydrogen Sulfide (H_2_S) (ppb) - Microbiological analysis (CFU/mL)	After 15 days	Deanship of Scientific Research at King Khalid University

*n*: number of patients; LLLT: low-level laser therapy; BTX-A: botulinum toxin type A; NR: Nor reported; TENS: transcutaneous electrical nerve stimulation; VAS: Visual Analogue.

**Table 2:** Main results of the included studies on the reduction in Hydrogen Sulfide (H_2_S) gas concentration measured in ppb (parts per billion).

Study/Year	aPDT	Tongue Scraper	Results/Estimated Effects
da Mota 2021 [44] After 7, 14 and 30 days	no numerical data provided	no numerical data provided	The authors reported no difference between groups (*p* > 0.05, *n* = 30)
do Vale 2020 [43] Immediately, mean	18.5	185.3	The authors reported a reduced H_2_S concentration in favour of aPDT (*p* = 0.003, *n* = 40)
After 7 days, mean	218.2	39.0	The authors reported a reduced H_2_S concentration in favour of aPDT (*p* = 0.000, *n* = 40)
Romero 2021 [24] Immediately, mean (SD)	68.3 (±68.0)	100.9 (±103.0)	Seems to have no difference between groups, but this results are imprecise (wide CI) MD −32.6 [95% CI −86.6 to 21.4]; *n* = 40; *p* = 0.24, very low-certainty evidence
After 7 days, mean (SD)	126.8 (±126.0)	123.1 (±126.0)	Seems to have no difference between groups, but this results are imprecise (wide CI) MD 3.7 [95% CI −67.6 to 75.0]; *n* = 40; *p* = 0.92, very low-certainty evidence
After 90 days, mean (SD)	152.5 (±176.8)	126.5 (±167.0)	Seems to have no difference between groups, but this results are imprecise (wide CI) MD 26.0 [95% CI −80.5 to 132.5]; *n* = 40; *p* = 0.63
Alshahrani 2020 [41] After 14 days, median (IQT)	42 (38)	65 (11.9)	The authors reported a reduced H_2_S concentration in favour of aPDT (*p* < 0.0001, *n* = 30)
Lopes 2015 [36] Immediately, median (IQT)	20 (20.2)	53 (7.0)	The authors reported a reduced H_2_S concentration in favour of aPDT (*p* = 0.008, *n* = 31)

ppb = parts per billion, H_2_S: hydrogen sulfide, aPDT = antimicrobial photodynamic therapy; SD: standard deviation; MD: mean difference; 95% CI: 95% confidence interval; IQT: interquartile.

**Table 3:** Summary of findings—GRADE approach.

Antimicrobial Photodynamic Therapy (aPDT) versus Tongue Scraper						
Population: patients diagnosed with halitosis Context: outpatient Intervention: aPDT Comparison: tongue scraper						
**Outcomes**	Anticipated Absolute Effects * **(95% CI)**		**Relative Effect** **(95% CI)**	**№ of Participants** **(Studies)**	**Certainty of the Evidence** **(GRADE)**	**Comments**
	**Risk with Tongue Scraper**	**Risk with aPDT**				
Hydrogen Sulfide (H_2_S) (in ppb) Assessed immediately	The mean H_2_S reduction was **100.9** ppb	MD **20.05 points higher** (53.22 lower to 93.22 higher)	**-**	40 (1 RCT)	⨁◯◯◯ VERY LOW ^a,b^	The evidence is very uncertain about the effect of aPDT on H2S reduction immediately and after 7 and 90 days of treatment. Additionally, 4 other studies seem to present a reduced H_2_S concentration in favour of aPDT. However, it was not possible to estimate the effect due to the lack of numerical data.
Hydrogen Sulfide (H_2_S) (in ppb) Assessed after 7 days	The mean H_2_S reduction was **123.1** ppb	MD 3.7 **points higher** (67.6 lower to 75 higher)	**-**	40 (1 RCT)	⨁◯◯◯ VERY LOW ^a,b^	
Hydrogen Sulfide (H_2_S) (in ppb) Assessed after 90 days	The mean H_2_S reduction was **126.5** ppb	MD **26 points higher** (80.5 lower to 132.5 higher)	**-**	40 (1 RCT)	⨁◯◯◯ VERY LOW ^a,b^	
Adverse events during the study	see comments	see comments	Not estimable	40 (1 RCT)	⨁◯◯◯ VERY LOW ^c,d^	No adverse events were reported in the aPDT group, and some participants reported discomfort and gagging sensation in the control group (no numerical data provided)

* **The risk in the intervention group** (and its 95% confidence interval) is based on the assumed risk in the comparison group and the **relative effect** of the intervention (and its 95% CI). CI: confidence interval; **RR:** risk ratio. **GRADE Working Group grades of evidence. High certainty:** we are very confident that the true effect lies close to that of the estimate of the effect. **Moderate certainty:** we are moderately confident in the effect estimate; the true effect is likely to be close to the estimate of the effect, but there is a possibility that it is substantially different. **Low certainty:** our confidence in the effect estimate is limited; the true effect may be substantially different from the estimate of the effect. **Very low certainty:** we have very little confidence in the effect estimate; the true effect is likely to be substantially different from the estimate of effect. **Explanations.** ^a^. Downgraded one level due to methodological limitation (incomplete outcome data). ^b^. Downgraded two levels for imprecision: only one study, very small number of participants, wide confidence interval. ^c^. Downgraded two levels due to methodological limitations (lack of information on allocation concealment and blinding of participants). ^d^. Downgraded one level for imprecision: only one study, very small number of participants and no events.

## Data Availability

Data extracted from included studies are available from the corresponding author at a reasonable request.

## Supplementary Materials

The following supporting information can be downloaded at: https://www.mdpi.com/article/10.3390/s22020469/s1, Table S1: Search strategies.
