# Supplementary material for: Effect of Photodynamic Therapy on Halitosis: A Systematic Review of Randomized Controlled Trials
Source: Sensors (Basel). 2022 Jan 8;22(2):469. doi: 10.3390/s22020469 (PMC8780921; doi:10.3390/s22020469)
Supplement: Supplementary file 1 [file sensors-22-00469-s001.zip › sensors-1512365-supplementary.pdf]

sensors MDPI

Supplementary Material

Table S1. Search strategies.

Database	Search strategy	Results
**MEDLINE (via Pubmed)**	#1 "Halitosis"[Mesh] OR Halitoses OR Malodour OR (Bad breath) OR (Breath odor) OR (Oral malodour) OR (fetor oris) OR (offensive-smelling breath) OR (fetor ex ore)<br>#2 "Photochemotherapy"[Mesh] OR (Photo Chemotherapies) OR (Photodynamic Therapy) OR (Therapy, Photodynamic) OR (Photodynamic Therapies) OR (Therapies, Photodynamic)<br>#3 "Low-Level Light Therapy"[Mesh] OR (Light Therapies, Low-Level) OR (Light Therapy, Low-Level) OR (Low Level Light Therapy) OR (Low-Level Light Therapies) OR (Therapies, Low-Level Light) OR (Therapy, Low-Level Light) OR (Photobiomodulation Therapy) OR (Photobiomodulation Therapies) OR (Therapies, Photobiomodulation) OR (Therapy, Photobiomodulation) OR LLLT OR (Laser Therapy, Low-Level) OR (Laser Therapies, Low-Level) OR (Laser Therapy, Low Level) OR (Low-Level Laser Therapies) OR (Laser Irradiation, Low-Power) OR (Irradiation, Low-Power Laser) OR (Laser Irradiation, Low Power) OR (Low-Power Laser Therapy) OR (Low Power Laser Therapy) OR (Laser Therapy, Low-Power) OR (Laser Therapies, Low-Power) OR (Laser Therapy, Low Power) OR (Low-Power Laser Therapies) OR (Low-Level Laser Therapy) OR (Low Level Laser Therapy) OR (Low-Power Laser Irradiation) OR (Low Power Laser Irradiation) OR (Laser Biostimulation) OR (Biostimulation, Laser) OR (Laser Phototherapy) OR (Phototherapy, Laser)<br>#4 #2 AND #3<br>#5 #1 AND #4	32
**Embase (via Elsevier)**	#1 'halitosis'/exp OR Halitoses OR Malodour OR (Bad breath) OR (Breath odor) OR (Oral malodour) OR (fetor oris) OR (offensive-smelling breath) OR (fetor ex ore)<br>#2 'photochemotherapy'/exp OR (Photo Chemotherapies) OR (Photodynamic Therapy) OR (Therapy, Photodynamic) OR (Photodynamic Therapies) OR (Therapies, Photodynamic)<br>#3 'low level laser therapy'/exp OR (Light Therapies, Low-Level) OR (Light Therapy, Low-Level) OR (Low Level Light Therapy) OR (Low-	54

	Level Light Therapies) OR (Therapies, Low-Level Light) OR (Therapy, Low-Level Light) OR (Photobiomodulation Therapy) OR (Photobiomodulation Therapies) OR (Therapies, Photobiomodulation) OR (Therapy, Photobiomodulation) OR LLLT OR (Laser Therapy, Low-Level) OR (Laser Therapies, Low-Level) OR (Laser Therapy, Low Level) OR (Low-Level Laser Therapies) OR (Laser Irradiation, Low-Power) OR (Irradiation, Low-Power Laser) OR (Laser Irradiation, Low Power) OR (Low-Power Laser Therapy) OR (Low Power Laser Therapy) OR (Laser Therapy, Low-Power) OR (Laser Therapies, Low-Power) OR (Laser Therapy, Low Power) OR (Low-Power Laser Therapies) OR (Low-Level Laser Therapy) OR (Low Level Laser Therapy) OR (Low-Power Laser Irradiation) OR (Low Power Laser Irradiation) OR (Laser Biostimulation) OR (Biostimulation, Laser) OR (Laser Phototherapy) OR (Phototherapy, Laser)<br>#3 #2 OR #3<br>#4 #1 AND #3	
**CENTRAL (via Wiley)**	#1 MeSH descriptor: [Halitosis] explode all trees<br>#2 Halitoses OR Malodour OR (Bad breath) OR (Breath odor) OR (Oral malodour) OR (fetor oris) OR (offensive-smelling breath) OR (fetor ex ore)<br>#3 MeSH descriptor: [Photochemotherapy] explode all trees<br>#6 (Photo Chemotherapies) OR (Photodynamic Therapy) OR (Therapy, Photodynamic) OR (Photodynamic Therapies) OR (Therapies, Photodynamic)<br>#4 MeSH descriptor: [Low-Level Light Therapy] explode all trees<br>#5 (Light Therapies, Low-Level) OR (Light Therapy, Low-Level) OR (Low Level Light Therapy) OR (Low-Level Light Therapies) OR (Therapies, Low-Level Light) OR (Therapy, Low-Level Light) OR (Photobiomodulation Therapy) OR (Photobiomodulation Therapies) OR (Therapies, Photobiomodulation) OR (Therapy, Photobiomodulation) OR LLLT OR (Laser Therapy, Low-Level) OR (Laser Therapies, Low-Level) OR (Laser Therapy, Low Level) OR (Low-Level Laser Therapies) OR (Laser Irradiation, Low-Power) OR (Irradiation, Low-Power Laser) OR (Laser Irradiation, Low Power) OR (Low-Power Laser Therapy) OR (Low Power Laser Therapy) OR (Laser Therapy, Low-Power) OR (Laser	29

	Therapies, Low-Power) OR (Laser Therapy, Low Power) OR (Low-Power Laser Therapies) OR (Low-Level Laser Therapy) OR (Low Level Laser Therapy) OR (Low-Power Laser Irradiation) OR (Low Power Laser Irradiation) OR (Laser Biostimulation) OR (Biostimulation, Laser) OR (Laser Phototherapy) OR (Phototherapy, Laser)<br>#7 #1 OR #2<br>#8 #3 OR #4 OR #5 OR #6<br>#9 #7 AND #8	
**LILACS/BBO (via BVS)**	#1 MH:Halitose OR Halitosis OR Halitosis OR C23.888.821.475<br>#2 MH:Fotoquimioterapia OR Photochemotherapy OR Fotoquimioterapia OR E02.186.500 OR E02.319.685 OR E02.774.722<br>#3 MH:(Terapia com Luz de Baixa Intensidade) OR (Low-Level Light Therapy) OR (Terapia por Luz de Baja Intensidad) OR E02.594.540 OR E02.774.500<br>#4 #2 OR #3<br>#5 #1 AND #4 AND (db:("LILACS"))	249
